# Posterior Decompression and Fusion: Whole-Spine Functional and Clinical Outcomes

**DOI:** 10.1371/journal.pone.0160213

**Published:** 2016-08-11

**Authors:** Anastasia Topalidou, George Tzagarakis, Konstantine Balalis, Alexandra Papaioannou

**Affiliations:** 1 Department of Orthopaedics and Traumatology, University Hospital of Heraklion, Faculty of Medicine, University of Crete, Heraklion, Greece; 2 Department of Anaesthesiology, University Hospital of Heraklion, Faculty of Medicine, University of Crete, Heraklion, Greece; University of Zaragoza, SPAIN

## Abstract

The mobility of the spine and the change in the angle of the curvatures are directly related to spinal pain and spinal stenosis. The aim of the study was the evaluation of morphology and mobility of the spine in patients who were subjected to decompression and posterior fusion with pedicle screws. The treatment group consisted of 20 patients who underwent posterior fixation of lumbar spine (one and two level fusion). The control group consisted of 39 healthy subjects. Mobility and curvatures of the spine were measured with a non-invasive device, the Spinal Mouse. Pain was evaluated with the Visual Analogue Scale (VAS). The Oswestry Disability Index (ODI) and the SF-36 were used to evaluate the degree of the functional disability and the quality of life, respectively. The measurements were recorded preoperatively and at 3, 6 and 12 months postoperatively. The mobility of the lumbar spine in the sagittal plane increased (p = 0.009) at 12 months compared to the measurements at 3 months. The mobility of the thoracic spine in the frontal plane increased (p = 0.009) at 12 months compared to the preoperative evaluation. The results of VAS, ODI and SF-36 PCS improved significantly (p<0.001). The levels of fusion exhibited a strong linear correlation (r = 0.651, p = 0.002) with the total trunk inclination in the upright position. Although pain, quality of life and spinal mobility in the sagittal and frontal planes significantly improved in the treatment group, these patients still had limited mobility and decreased curves/angles values compared to control group.

## Introduction

Spinal stenosis presents with symptoms related to the anatomical reduction of the diameter of the spinal canaland the neural foramina [1–5]. It is classified as primary, caused by congenital abnormalities and secondary, caused by degenerative changes, infections, traumas, tumours or intra/postoperative changes. Decompressive laminectomy and fixation is the accepted treatment of spinal stenosis [2]. Spinal fixation is recommended for the treatment of instability [2,5–7], whereas effective limitation of abnormal motion reduces pain [8,9].

Spinal mobility and changes in spinal curvatures are related to both spinal stenosis and pain reported by the patient [10,11,12]. Several methods have been developed for the evaluation of the mobility of the spine and its curvatures. Although dynamic plain radiographs provide an excellent mean for segmental recording, the risk of radiation limits their role for follow-up examinations and routine examinations in young patients [11,13]. Moreover, plain radiograph provides imaging of only parts of the spine. Imaging of the entire spine results in significantly higher radiation burden [14]. Magnetic Resonance Imaging (MRI) is an alternative but its use is limited by high cost and the inability to dynamically examine the spine in conventional MR scanners [15]. Other techniques applied for the evaluation of the spinal surface, such as the scoliometer, the kyphometer [16], the flexible curve, the goniometer, the inclinometer [17], the measurement of fingertip to floor distance, the Shober index [18], the tape measurement method [19], the electromagnetic probe [20] and the fiber-optic method [11], either have a poor reliability and validity or they are time-consuming. At the same time, most of these methods can measure only one part of the spine at a time [17,18] often providing limited information [21]. In addition, some methods have limited recording potential of only the sagittal plane or the frontal plane and not both of them at the same setting [11].

This study used the Spinal Mouse, a new non-invasive method that is valid, reliable and safe. It also has low cost per examination, requires limited time for evaluation, provides recording potential of both the sagittal and the frontal plane and finally has the ability to perform multiple clinical tests [22–24].

The aim of the present study was the evaluation of spinal morphology and mobility in patients with spinal stenosis subjected to decompression and posterior lumbar fusion with pedicle screws. The null hypothesis was that the average postoperative functional improvement would be the same as the average improvement during the 0–3, 0–6, 0–12, 3–6, 3–12 and 6–12 months postoperatively.

## Materials and Methods

### Participants

From September 2010 to January 2012 all patients who underwent posterior fusion with pedicle screws and rods for spinal stenosis were recruited to the study. The diagnosis of spinal stenosis was made clinically and was confirmed with MRI. The exclusion criteria included: previous spinal surgery, leg length discrepancy (LLD), total hip arthroplasty or hip dysplasia, other scheduled surgery on the spine or the lower limbs in the next twelve months, spinal metastasis or spinal tumour, Alzheimer’s disease, Parkinson’s disease, ankylosing spondylitis, spondyloarthropathy, paraparesis or lower extremity paresis, hemiplegia or stroke, movement disorders, psychiatric disease, age higher than 75 years or living outside the island of Crete. All patients were operated on by the same orthopaedic surgeon andwere followed-up for one year postoperatively.

The Control Group (CG) consisted of 39 healthy subjects with no history of neuromuscular and musculoskeletal pathology or injury of the spine and/or lower limps.

All participants were informed in detail for the purpose of the study and provided written consent according to the Bioethics Committee of the University Hospital of Heraklion, Greece. The Scientific Committee of the University Hospital of Heraklion, Greece, approved the study (10787/20-12-10).

### Method

The curvature and the mobility of the spine of both groups were evaluated with the Spinal Mouse ® (Idiag, Volkerswill, Switzerland), a computer-assisted wireless telemetry device. This portable device is guided along the spinous processes of the vertebral column. The values are transferred in real time to a computer which reproduces a two-dimensional graph of the spine [25]. The recording frequency is 150Hz.

In the Treatment Group (TG), the evaluation of the spine with the Spinal Mouse and the questionnaires completion were made preoperatively on the day of the hospital admission. Follow-up measurements were performed in 3, 6 and 12 months postoperatively (±one calendar week).

In the CG, spinal function alone was evaluated only once at the same environment.

The technique and the measured parameters have been described in the literature [25].

Furthermore, the subjects of the TG were asked to fill three questionnaires. Lower-back pain (VAS-back) and leg pain (VAS-leg) was evaluated with the Visual Analogue Scale (VAS) where zero (0) represents no pain at all and ten (10) represents the worst pain possible [26]. The Oswestry Disability Index (ODI) was used for the evaluation of functional disability [27]. The health-related quality of life was assessed with the SF-36 and the Physical Component Summary (PCS) and the Mental Component Summary (MCS) scores were recorded [28].

### Statistical analysis

Paired t-test and repeated measures analysis of variance (ANOVA) with Bonferroni pot-hoc test were used to test whether there was a significant surgery effect on Spinal Mouse’s parameters at 3, 6 and 12 months postoperatively. The same analysis was used to test whether there was a significant surgery effect on SF-36, ODI and VAS.

The relationship between all Spinal Mouse parameters and the results from the questionnaires SF36, ODI, VAS postoperatively at 12 months, with fusion levels, was tested through Pearson’s correlation coefficient.

ANOVA was used to determine whether there were any significant differences between the means of CG and TG 12 months postoperatively, based on Spinal Mouse’s measurements.

A Receiver Operating Characteristic (ROC) analysis was used to determine the optimal cut-off differential entropy point for the separation of patients and control subjects on all Spinal Mouse’s parameters. SPPS 15.0 was used for statistical analysis. All statistical tests were carried at the 5% level of significance.

## Results

During the study period 45 patients (n = 27 males and n = 18 females) were initially recruited. Thirty-eight patients successfully completed the re-evaluation process according to the study protocol. Thirty patients underwent lumbar spinal fusion, 3 patients underwent thoracic spinal fusion and 5 patients underwent thoracolumbar spinal fusion. The levels of the fusion ranged from one-level fusion (two vertebrae) to nine-level fusion (ten vertebrae). In order to have an homogeneous TG group, only patients subjected to one or two levels lumbar fusion were included in the analysis. The demographic and somatometric characteristics of the TG and the CG are presented in Table 1. There were no statistically significant differences between the two groups.

**Table 1:** Demographic characteristics of the participants of the Treatment Group (TG) and the Control Group (CG).

		Treatment Group (TG) n = 20	Control Group (CG) n = 39
Gender	Male	n = 14 (70%)	n = 17 (43.6%)
	Female	n = 6 (30%)	n = 22 (56.4%)
Age		56.40 (±12.47)	50.67 (±11.18)
Height		1.69 (±0.08)	1.69 (±0.07)
Weight		79.20 (±17.53)	73.51 (±13.13)
BMI		27.56 (±4.94)	25.72 (±3.50)

### Spine curvatures sagittal plane

#### Upright position

There were no statistically significant changes for the TG.

#### Full flexion and full extension

There was no statistically significant difference in these positions regarding the thoracic and the lumbar curves during the re-evaluations. However, the hip-sacral angle (Sac_Hip) and the overall trunk inclination (Incl) exhibited a statistically significant increase at the 6-month and the 12-month re-evaluations.

### Spine curvatures frontal plane

#### Upright position and Right lateral flexion

There was no statistically significant difference in any parameter in these positions.

#### Left lateral flexion

There were no statistically significant differences in the evaluations of lumbar curve, Sac_Hip and Incl. However, thoracic curve exhibited a statistically significant increase at 12 months postoperatively compared to preoperative evaluation.

The results of all measurements performed in each position, all the parameters studied in sagittal and frontal plane and the statistically significant changes are presented in Table 2.

**Table 2:** Spine curvatures measurements for all positions in sagittal and frontal plane of the patients.

			Spinal Curvatures			
			Preoperatively (mean value and SD)	3 months (mean value and SD)	6 months (mean value and SD)	12 months(mean value and SD)
**Sagittal Plane**	**Upright Position**	Sac_Hip	12.25^ο^±1.38^ο^	9.30^ο^+1.38^ο^	9.35^ο^±2.29^ο^	9.40^ο^±2.23^ο^
		Thoracic curve	40.60^ο^±3.06^ο^	40.55^ο^±3.06^ο^	43.60^ο^±2.43^ο^	47.00^ο^±2.46^ο^
		Lumbar curve	-20.90^ο^±1.92^ο^	-17.05^ο^±1.88^ο^	-18.95^ο^±2.26^ο^	-21.40^ο^±1.98^ο^
		Trunk inclination	3.20^ο^±1.10^ο^	3.75^ο^±0.91^ο^	3.10^ο^±1.07^ο^	2.60^ο^±1.44^ο^
	**Full Flexion**	Sac_Hip	36.65^ο^±3.82^ο^	39.90^ο^±2.28^ο^	52.65^ο^±3.78 **¥** **p<0.031**	65.25^ο^±3.41^ο^ * **p<0.000,** **¥** **p<0.000**
		Thoracic curve	52.35^ο^±2.18^ο^	56.60^ο^±2.85^ο^	57.50^ο^±3.81^ο^	56.75^ο^±2.88^ο^
		Lumbar curve	2.00^ο^±3.13^ο^	0.25^ο^±1.95^ο^	2.45^ο^±2.12^ο^	5.05^ο^±2.24^ο^
		Trunk inclination	51.10^ο^±5.33^ο^	54.75^ο^±3.21^ο^	70.05^ο^±4.47^ο^	85.20^ο^±3.64^ο^ * **p<0.000,** **¥** **p<0.000**
	**Full Extension**	Sac_Hip	5.90^ο^±1.96^ο^	3.35^ο^±1.66^ο^	-0.15^ο^±2.05^ο^	-0.75^ο^±1.99^ο^
		Thoracic curve	30.70^ο^±3.11^ο^	30.25^ο^±3.21^ο^	30.00^ο^±2.72^ο^	28.65^ο^±3.64^ο^
		Lumbar curve	-21.45^ο^±2.30^ο^	-19.90^ο^±2.20^ο^	-20.25^ο^±2.05^ο^	21.90^ο^±2.18^ο^
		Trunk inclination	-6.65^ο^±1.68^ο^	-8.30^ο^±1.58^ο^	-11.30^ο^±1.97^ο^	-13.55^ο^±1.99^ο^ **¥** **p = 0.016**
**Frontal Plane**	**Left Lateral Flexion**	Sac_Hip	-3.26^ο^±0.68^ο^	-2.47^ο^±0.79^ο^	-5.06^ο^±0.70^ο^	-6.07^ο^±1.10^ο^
		Thoracic curve	22.12^ο^±2.48^ο^	26.54^ο^±2.68^ο^	30.11^ο^±2.80^ο^	34.36^ο^±3.11^ο^ * **p = 0.018**
		Lumbar curve	9.06^ο^±0.93^ο^	9.32^ο^±1.77^ο^	9.49^ο^±0.93^ο^	10.72^ο^±1.47^ο^
		Trunk inclination	15.55^ο^±1.26^ο^	15.45^ο^±1.49^ο^	18.96^ο^±1.16^ο^	22.16^ο^±2.25^ο^
	**Upright Position**	Sac_Hip	0.46^ο^±0.67^ο^	2.39^ο^±1.11^ο^	-0.46^ο^±0.71^ο^	-0.19^ο^±0.75^ο^
		Thoracic curve	-3.78^ο^±0.86^ο^	0.02^ο^±4.06^ο^	-2.59^ο^±1.26^ο^	-3.03^ο^±0.74^ο^
		Lumbar curve	2.56^ο^±0.68^ο^	3.07^ο^±0.70^ο^	2.52^ο^±0.69^ο^	2.02^ο^±0.62^ο^
		Trunk inclination	1.09^ο^±0.63^ο^	2.34^ο^±1.96^ο^	1.75^ο^±0.49^ο^	1.54^ο^±0.54^ο^
	**Right Lateral Flexion**	Sac_Hip	6.01^ο^±1.14^ο^	4.02^ο^±1.19^ο^	3.76^ο^±1.12^ο^	6.15^ο^±1.32^ο^
		Thoracic curve	-27.76^ο^±2.27^ο^	-27.94^ο^±3.95^ο^	-32.29^ο^±1.73^ο^	-30.90^ο^±3.06^ο^
		Lumbar curve	-4.51^ο^±1.32^ο^	-7.47^ο^±1.31^ο^	-7.50^ο^±1.12^ο^	-8.40^ο^±1.25^ο^
		Trunk inclination	-15.16^ο^±1.09^ο^	-16.26^ο^±1.25^ο^	-17.41^ο^±1.29^ο^	-20.03^ο^±1.56^ο^

Statistically significant difference

* compared with the preoperative evaluation and

¥ compared with the 3 months evaluation.

### Spinal mobility

#### Sagittal plane

Although the changes in the mobility of the thoracic spine were not statistically significant, it is worth mentioning that the total ROM of the thoracic spine from full bending to full extension increased from 21.35^ο^±3.26^ο^ preoperatively to 24.20^ο^±4.00^ο^ 12 months postoperatively. Regarding the ROM of the lumbar spine from the upright position to the full flexion, a statistically significant increase of the ROM was observed 12 months postoperatively (17.35^ο^±1.86^ο^ at 3 months, 21.75°±2.36^ο^ at 6 months, 26.40^ο^±2.68^ο^ at 12 months). The statistically significant changes in the mobility mainly concerned the mobility of the Sac_Hip which exhibited a continuous increase. The changes in the mobility of the Sac_Hip cumulatively with the small changes in the mobility of the thoracic and the lumbar spine exhibited a statistically significant increase in the Incl.

#### Frontal plane

There were no statistically significant changes in the ROM of the Sac_Hip, Incl and lumbar spine in any type of measurements. Only thoracic spine presented a statistically significant increase in its ROM at 12 months (37.67^ο^±2.96^ο^) compared to preoperative assessment (25.90^ο^±2.53^ο^).

The statistically significant changes that were recorded in the sagittal and the frontal plane are presented in Table 3.

**Table 3:** Statistically significant changes in the mobility of the spine in the sagittal and frontal plane among re-evaluations.

			Spinal Mobility				
			6 vs pre	12 vs pre	6 vs 3	12 vs 3	12 vs 6
**Sagittal Plane**	**AF** 95% CI	Sac_Hip	p = 0.017 (2.484, 35.816)	p<0.000 (17.886, 45.014)	p = 0.026 (1.108, 25.192)	p<0.000 (13.101, 37.799)	
		Lumbar curve				p = 0.009 (1.753, 16.347)	p = 0.025 (0.415, 8,885)
		Trunk inclination		p<0.000 (15.254, 54.646)	p = 0.036 (0.698, 31.502)	p<0.000 (17.389, 45.711)	
	**AE** 95% CI	Trunk inclination		p = 0.030 (0.422, 12.478)		p = 0.033 (0.236, 8.264)	
	**FE** 95% CI	Sac_Hip	p = 0.026 (1.884, 42.816)	p<0.000 (19.490, 50.910)	p = 0.031 (1.023, 31.977)	p<0.000 (17.019, 41.681)	
		Trunk inclination		p<0.001 (17.571, 64.929)		p<0.001 (20.171, 51.429)	
**Frontal Plane**	**SL** 95% CI	Thoracic curve		p = 0.009 (2.220, 21.310)			

Sagittal Plane: The AF represents the mobility of the spine during the shift from the upright position to the full flexion. ΑΕ: mobility of the spine during the shift from the upright position to the full extension. FE: the total ROM from the full bending to the full extension.

Frontal plane: SL: mobility of the spine during the shift from the standing position to the full left lateral bending.

(6 vs pre = 6 months versus preoperative measurement, 6 vs 3 = 6 months versus 3 months, 12 vs pre = 12 months versus preoperative, 12 vs 3 = 12 months versus 3 months and 12 vs 6 = 12 months versus 6 months)

### Comparison of the CG with the TG

All the parameters of the Spinal Mouse of the TG that were recorded in the final evaluation at 12 months postoperatively were compared with the corresponding parameters of the CG.

#### Sagittal plane

The TG displayed significantly lower mobility compared to the CG at all measured mobility parameters. Same results, with the TG presenting statistically significant lower values than CG were observed for Sac_Hip in upright position (p = 0.002), full extension (p = 0.001) and in the ROM from the upright position to full flexion (p = 0.008) and from full bending to full extension (p = 0.005). Also, CG presented statistically significant greater ROM from full bending to full extension (p = 0.04). No significant differences were observed in the thoracic spine.

#### Frontal plane

In the frontal plane, the TG displayed significantly lower mobility compared to the CG at all measured mobility parameters. Also, there were statistically significant differences between the two groups in the upright position for the Sac_Hip p = 0.004, the thoracic curve p = 0.001 and the Incl p = 0.02. The Incl exhibited statistically significant differences in the full right lateral bending p = 0.003, in the ROM from the standing position to the full right lateral bending p = 0.018 and from the full left lateral bending to the full right lateral bending p = 0.039. Thoracic curve was statistically significantly greater for CG in comparison with TG (p = 0.043) only in full left lateral bending. The other eleven parameters did not exhibit statistically significant differences.

### Questionnaires

#### SF-36 PCS and SF-36 MCS

There were statistically significant improvements between all the re-evaluations for the physical condition, but no differences for mental condition were observed.

#### ODI

The evaluation of the results regarding the functional disability showed that all re-evaluations exhibited a statistically significant reduction in the score and therefore an improvement in the condition of the participants.

#### VAS-back and VAS-leg

Similarly, the analysis of the results on the lower-back pain and leg pain exhibited a statistically significant reduction among all the re-evaluations.

The mean values and the statistical significant changes for all questionnaires are presented in Table 4.

**Table 4:** Statistically significant improvements from the evaluation of the questionnaires SF-36 PCS, SF-36 MCS, ODI, VAS-back and VAS-leg.

		SF-36 PCS 95% CI	SF-36 MCS 95% CI	ODI 95% CI	VAS-back 95% CI	VAS-leg 95% CI
**Mean value and SD**	preoperative	23.34±1.34	53.86±2.26	76.20%±3.83%	7.65±0.38	8.45±0.48
	3 months	34.99±1.17	58.04±1.33	49.75%±3.50%	3.40±0.36	3.75±0.46
	6 months	46.63±1.69	60.18±1.18	26.15%±2.52%	1.25±0.37	1.60±0.37
	12 months	52.37±1.68	60.53±1.18	14.90%±2.33%	0.40±0.22	0.80±0.25
**Comparison of re-evaluations**	3 months vs. preoperative	p<0.001* (7.294, 16.006)	p = 0.199 (1.039, 9.399)	p< 0.001* (19.016, 33.884)	p<0.001* (3.333, 5.167)	P<0.001* (3.748, 5.652)
	6 months vs. preoperative	p<0.001* (17.936, 28.634)	p = 0.330 (2.406, 15.046)	p<0.001* (37.805, 62.295)	p<0.001* (4.973, 7.827)	p<0.001* (5.463, 8.237)
	12 months vs. preoperative	p<0.001* (23.745, 34.315)	p = 0.403 (2.949, 16.289)	p<0.001* (47.614, 74.986)	p<0.001* (6.056, 8.444)	p<0.001* (6.244, 9.056)
	6 months vs. 3 months	p<0.001* (7.597, 15.673)	p = 1.00 (-2.459, 6.739)	p<0.001* (14.557, 32.643)	p<0.001* (1.088, 3.212)	p<0.001* (1.377, 2.923)
	12 months vs. 3 months	p<0.001* (13.599, 21.161)	p = 1.00 (-3.065, 8.045)	p<0.001* (23.950, 45.750)	p<0.001* (1.970, 4.030)	p<0.001* (1.908, 3.992)
	12 months vs. 6 months	p<0.001* (3.430, 8.060)	p = 1.00 (-1.400, 2.100)	p<0.001* (6.154, 16.346)	p<0.046* (0.011, 1.689)	p = 0.013* (0.125, 1.475)

### Fusion levels

In the sagittal plane a strong linear correlation (0.5≤|r|<0.7) was demonstrated between the Incl in upright position and the spinal levels that were fused (r = 0.651, p = 0.002). Also, the ROM of lumbar spine from the upright position to the full flexion showed a moderate negative linear correlation (0.35≤|r|<0.5), with fusion levels (r = -0.491, p = 0.028).

All parameters in frontal plane exhibited a weak linear correlation compared to the levels of fusion (|r|<0.3).

### Diagnostic value of the method

Series of cut-off values were created by selecting midpoints through the order of the Spinal Mouse values. For each cut-off value, (1 − specificity) and (sensitivity) values are computed as percentages based on the true positives and false negatives. Also, the abovementioned figures present the coordinates of each cut-off value in the 2D space of (1 − specificity) and (sensitivity). Each point has a distance from the diagonal (the non-discrimination line). The point with the greater distance from the diagonal was selected and it indicates the optimum trade-off between the true positives and the false negatives Spinal Mouse values (δ = [(1-specificity)-sensitivity] / sqrt (2)).

**Fig 1:**
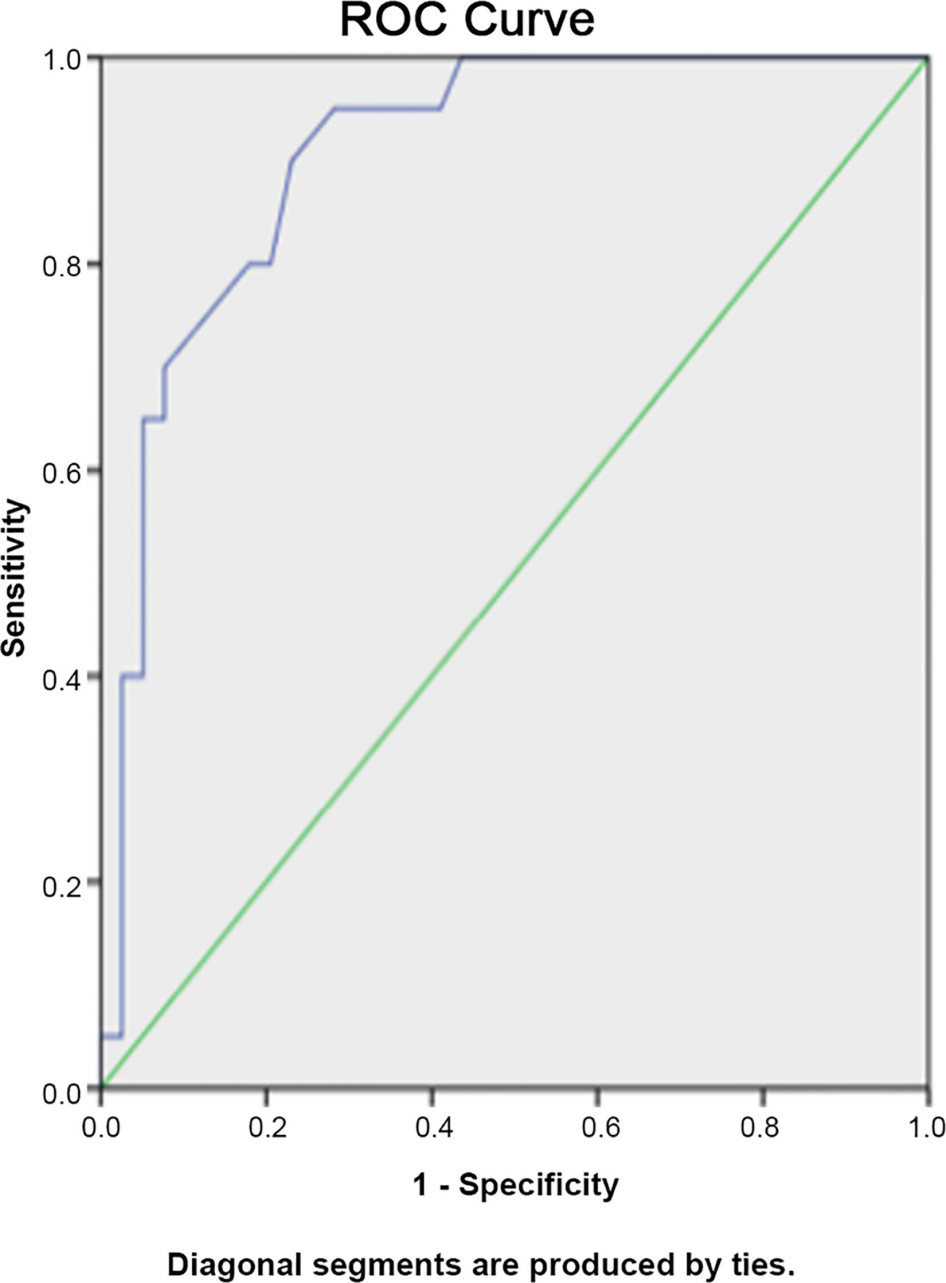
Receiver Operating Characteristic (ROC) curves: True positive rates (sensitivity) and false positive rates (1 − specificity) for the curvature of lumbar spine in full extension measurement in the sagittal plane.

Fig 1 shows the ROC analysis. Table 5 presents the ROC analysis’ results.

**Table 5:** The results of ROC analysis.

	Measurements	Optimal cut-off value (^ο^)	Sensitivity (%)	Specificity (%)	AUC (%)
**Sagittal Plane**	Upright position, curvature of lumbar spine	-27.5	85.0	69.2	80.7
	Full extension, curvature of lumbar spine	-33.5	80.0	82.1	90.6
	Full extension, total trunk inclination (Incl)	-15.5	80.0	74.4	82.0
	Mobility (ROM) of lumbar spine from upright position to full extension	-4.5	90.0	69.2	79.7
	Mobility (ROM) for total trunk inclination (Incl) from upright position to full extension	10.5	70.0	74.4	80.6
**Frontal Plane**	Lumbar curve in right lateral bending	-9.35	75.0	76.9	83.5
	Total trunk inclination (Incl) in right lateral bending	-23.5	80.0	79.5	88.5
	Mobility (ROM) for total trunk inclination from upright position to full left lateral bending	-21.4	80.0	79.5	87.1
	Mobility (ROM) of lumbar spine from upright position to full right lateral bending	-11.35	80.0	79.5	86.9
	Mobility (ROM) of lumbar spine from full left lateral bending to full right lateral bending	-15.15	70.0	97.4	89.6

## Discussion

Increased life expectancy results in more people experiencing back pain and spinal stenosis, a common indication for spine surgery. Currently, increased attention is paid regarding mobility and functionality of the spine and their correlation with back pain and health status [29,30]. Many medical devices and diagnostic tools have been developed for the evaluation of the spine. The regular follow ups, the need for evaluation of the spine as a whole and the need for recording of morphological and mobility data, requires the use of a valid, reliable and non-invasive method. The present study used a relatively new tool that provided imaging of the spine from C7 to S2-S3 in order to evaluate the functional and morphological changes of the spine in patients who underwent posterior lumbar fusion with pedicle screws and robs.

### Spine curvatures sagittal plane

The preoperative lumbar hypolordosis remained <20^ο^ at 3 and 6 months postoperatively and slightly increased to 21.40^ο^±1.98^ο^ at 12 months. The lumbar hypolordosis is probably caused by a combination of factors. First, it is known that the aging of the spine is associated with a reduction in the lumbar lordosis (LL) [31] and the participants in the present study were individuals with an average age of 56.40±12.47 years. Moreover, the paraspinal muscle spasm, which is observed in individuals with back pain and those with a history of spondylosis, is the cause of significant hypolordosis [32]. These two factors can explain the reduced preoperative LL (-20.90^ο^±1.92^ο^) which was further reduced at 3 months (-17.05^ο^±1.88^ο^) and remained almost stable at 6 months (-18.95^ο^±2.26^ο^). The course of the initial reduction and then the increase in the lordosis is also reported by other researchers who examined the posterior lumbar fusion [33]. However, the fact that the LL was just -21.40^ο^±1.98^ο^ even at 12 months postoperatively, is the result of the use of a stabilization system with rods in the applied-technique [4,33]. Characteristically, Boos and Webb (1997) [4] reported that the use of rods reduces the LL especially if the fusion includes healthy motion segments.

The thoracic kyphosis (TK) was found within normal limits exhibiting a gradual increase from 40.60^ο^±3.06^ο^ preoperatively to 47.00^ο^±2.46^ο^ at 12 months postoperatively. Chaleat-Valayer et al. (2011) [34] reported that the average value of the thoracic curvature in healthy subjects with no spinal disorders is 50.1^ο^±10.4^ο^ whereas the angle of the kyphosis in individuals with low back pain is reduced at an average of 46.7^ο^±18.3^ο^. Similarly, the average of the TK was reported low with an average of 36.9^ο^±8.9^ο^ on individuals with low back pain [10].

### Spine curvatures frontal plane

Most studies explore the spine in the sagittal plane, while studies exploring the frontal plane focused mainly in scoliosis [35,36]. To the best of our knowledge, no study has been found to explore the frontal plane of the spine of individuals who have undergone any kind of spinal surgery.

The limited improvement observed in the lateral thoracic and lumbar curvatures in the upright position was probably caused by the postoperative reduction of the pain and the elimination of the muscle spasms. Degeneration of the spine, back or radicular pain and the impaired equilibrium, result in a progressive pattern of deviation of the spine from the anatomic configuration of its curvatures [37]. Regarding the lateral flexions of the torso, there was a significant increase in the thoracic curvature but only for the left lateral flexion at 12 months. On the contrary, there was no statistically significant improvement of the right lateral flexion. This could be due to the fact that preoperatively the studied patients had a smaller left thoracic lateral flexion in comparison with the right one. This difference is possibly the result of the equalizing position of the torso due to pain and consequently the angle of the motion of the area opposite the suffering area was reduced. However, since there were no recordings in the present study of the projection of the hernias and of the most painful site, safe results cannot be concluded.

### Spinal mobility

There were no statistically significant changes in the ROM of the thoracic curvature in the sagittal plane during re-evaluations. Lumbar curvature presented an increase in mobility from upright position to full bending at 12 months postoperatively (26.40^ο^±2.68^ο^) compared to the measurements at 3 (17.35^ο^±1.86^ο^) and 6 months (21.75^ο^±2.35^ο^), but not with the preoperative assessment (23.15^ο^±3.06^ο^). There are no other studies in the literature that examine the ROM of the thoracic and the lumbar spine separately in patients who underwent posterior fusion. Studies with different surgical techniques explored the changes in the curvatures (lordosis and kyphosis) and not the mobility of these levels [5,9,7,12]. A study, in which the ROM of a dynamic stabilization level, the ROM of every adjacent segment and the total lumbar ROM of patients who underwent a multi-level posterior dynamic stabilization were examined with the use of lateral flexion/extension radiographs, reported that the ROM of the total lumbar spine and the instrumented levels exhibited a statistically significant reduction postoperatively, whereas the ROM of the superjacent and the subjacent levels of the instrumentation displayed a non-significant increase [33]. In general, spinal fixation does not seem to significantly modify the mobility of the thoracic and the lumbar spine.

The small reduction in all mobility assessments that is statistically insignificant and presented in the ROM of the lumbar spine3 months postoperatively, can be explained by the use of the brace worn by the patients for 3 months on average postoperatively. Park et al (2009) [33] reported that the measurements of the ROM were more reliable after 6 months postoperatively and onwards because the use of the back brace had a negative result on the ROM.

The statistically significant increases of the ROM of the Sac_Hip and Incl in almost all measurements of mobility can be explained by the improvement of the sciatica. Initially, the preoperative VAS score for the lower extremities of the patients was greater than the respective one for back pain. This exhibited a statistically significant and rapid improvement postoperatively. This improvement of the radicular pain resulted in an improvement in the functionality and the mobility of the hip-sacral area. The improvement in the quality of life, the mobility and the functionality of the torso as a result of the reduction of radicular pain has been proven [38].

Finally, as to the frontal plane, there were no significant changes in most of the mobility parameters. The only statistically significant difference, presented in the thoracic spine was probably caused by two factors. Firstly, is the postoperative elimination of the one-sided muscle spasm and therefore the counter balance of the forces that act on the spine and secondly, the elimination of pain and therefore the improvement in the ROM of the suffering area [32,39].

### Diagnostic value of the method

The ROC analysis showed that some of the parameters recorded by the Spinal Mouse, such as the lumbar curvature in full extension and the mobility of lumbar spine from full left lateral bending to full right lateral bending, have a diagnostic ability that is similar to the Myelography, CT and MRI, as shown in Table 6 [40–45]. The Spinal Mouse method is characterized by satisfactory diagnostic ability, reproducibility and low cost, whereas it is easy to use and non-invasive, allowing the recording of spinal mobility.

**Table 6:** Typical values of sensitivity and (1 − specificity) of myelography, computed tomography (CT), MRI and spinal mouse.

	Myelography	CT	MRI	Spinal Mouse									
				Sagittal Plane					Frontal Plane				
				Full extension, curvature of lumbar spine	Upright position, curvature of lumbar spine	Full extension, total trunk inclination (Incl)	Mobility (ROM) of lumbar spine from upright position to full extension	Mobility (ROM) for total trunk inclination (Incl) from upright position to full extension	Mobility (ROM) of lumbar spine from full left lateral bending to full right lateral bending	Mobility (ROM) of lumbar spine from upright position to full right lateral bending	Mobility (ROM) for total trunk inclination from upright position to full left lateral bending	Lumbar curve in right lateral bending	Total trunk inclination (Incl) in right lateral bending
**Sensitivity**	0.77–0.78	0.77–0.88	0.81–0.97	0.80	0.85	0.80	0.90	0.70	0.70	0.80	0.80	0.75	0.80
**1-specificity**	0.28	0.17–0.20	0.00–0.06	0.18	0.30	0.25	0.30	0.25	0.03	0.20	0.20	0.23	0.20

### Comparison of the CG with the TG and fusion levels

The comparison between the TG results at 12months and the CG measurements showed that the lumbar spine and Sac_Hip exhibited significant differences regarding the mobility and the angles of the curvatures both in the sagittal and the frontal plane. The values of the TG were noticeably lower, demonstrating that the posterior pedicle screw fixation reduced the lumbar motion ability due to the stabilization and the immobilization of the vertebral segments, whereas the LL was lower. A reduced LL is proportionally associated with a reduced Sac_Hip and therefore the Sac_Hip values appeared significantly lower for TG as well. The use of rods in the technique of the stabilization is the reason for the reduction of the LL and the ROM. This reduction is even greater when healthy motion segments are required in the fusion. In fact, the more the levels of the fusion are, the more these factors are reduced [4]. Even in our study, where we examined only one and two level fusion, the ROM of lumbar spine from upright position to full flexion and the Incl in upright position exhibited a linear correlation with the fusion levels. Therefore the use of spinal fixation in the treatment of spinal stenosis should be limited to the fewest possible levels in order to avoid the stabilization of healthy motion segments and have a result that is as functional as possible regarding morphology as well as mobility. Moreover, an effort should be made to achieve as normal lordosis as possible. It is worth mentioning that decompression with instrumented fusion exhibits better results postoperatively in comparison with decompression with no use of stabilization materials. Also, the use of rods did not seem to cause changes in functionality compared with other stabilization methods and each method seems to have each own specific disadvantages [46]. Therefore, both the restoration of the LL, which is reduced preoperatively as shown in the present study, and the involvement of the fewest possible levels in the stabilization should be taken into consideration.

### Questionnaires

The assessment of the questionnaires showed gradually increasing and significant improvements in progressive time among all the re-evaluations regarding SF-36 PCS, ODI, VAS-back and VAS-leg. Only SF-36 MCS did not show any significant differences. This means that the physical condition, the degree of the functional disability and the perception of the pain are factors which improved significantly after surgery. The VAS-leg had a greater score than the VAS-back preoperatively in agreement with another study which used the dynamic stabilization system [33]. Although studies report that significant improvements of pain are observed only in cases where the dynamic stabilization is used [5,9,47], the present study showed a greater improvement of pain. Further studies should explore longitudinally the above discrepancy.

There are several studies also reporting improvement of ODI, SF -36 PCF and SF-36 MCF [48–50]. The fact that in the present study no significant improvement at SF-36 MCF was observed could be due to the relatively high preoperative score of the recruited patients. On the other hand, in a study examining the anterior-posterior fusion for low-grade isthmic spondylolisthesis in 23 patients no statistically significant improvements were found postoperatively regarding the SF-36 MCS [51].

Although a limitation of the present study was the small size of the final TG, however the subjects were relatively homogeneous (only lumbar spine pedicle screws and rods fixation and one or two level fusion, by the same surgeon), the results reflect the essence of patients’ postoperative condition.

## Conclusion

Treatment of spinal stenosis with decompression and posterior fusion with pedicle screws and rods exhibits good results regarding pain and satisfaction of patients who obtain early physical and functional recovery. The hypolordosis that is present preoperatively may persist postoperatively with slight improvement. Nevertheless, the whole of the spine seems to act in an equalizing manner through the angles that are formed in the Sac_Hip and the thoracic curvature, creating a structure which can absorb any force that is applied and it can function with no pathological or functional problems. The individuals who undergo posterior fusion display worse angles, curves and ROM of the lumbar spine and Sac_Hip in comparison with the healthy population but this does not seem to affect the subjective evaluation of their condition based on the questionnaires. Finally, this non-invasive method seems to have a very good diagnostic ability regarding the differentiation between the individuals with spinal stenosis and the healthy individuals.
